# Analysis of expression sequence tags from a full-length-enriched cDNA library of developing sesame seeds (*Sesamum indicum*)

**DOI:** 10.1186/1471-2229-11-180

**Published:** 2011-12-24

**Authors:** Tao Ke, Caihua Dong, Han Mao, Yingzhong Zhao, Hong Chen, Hongyan Liu, Xuyan Dong, Chaobo Tong, Shengyi Liu

**Affiliations:** 1 Key Laboratory for Oil Crops Biology, The Ministry of Agriculture, PR China. Oil Crops Research Institute, Chinese Academy of Agricultural Sciences, No.2 Xudong Second Road, Wuhan 430062, China; 2 Department of Life Science and Technology, Nanyang Normal University, Wolong Road, Nanyang 473061, China

## Abstract

**Background:**

Sesame (*Sesamum indicum*) is one of the most important oilseed crops with high oil contents and rich nutrient value. However, genetic improvement efforts in sesame could not get benefit from molecular biology technology due to poor DNA and RNA sequence resources. In this study, we carried out a large scale of expressed sequence tags (ESTs) sequencing from developing sesame seeds and further conducted analysis on seed storage products-related genes.

**Results:**

A normalized and full-length enriched cDNA library from 5 ~ 30 days old immature seeds was constructed and randomly sequenced, leading to generation of 41,248 expressed sequence tags (ESTs) which then formed 4,713 contigs and 27,708 singletons with 44.9% uniESTs being putative full-length open reading frames. Approximately 26,091 of all these uniESTs have significant matches to the counterparts in Nr database of GenBank, and 21,628 of them were assigned to one or more Gene ontology (GO) terms. Homologous genes involved in oil biosynthesis were identified including some conservative transcription factors regulating oil biosynthesis such as LEAFY COTYLEDON1 (LEC1), PICKLE (PKL), WRINKLED1 (WRI1) and majority of them were found for the first time in sesame seeds. One hundred and 17 ESTs were identified possibly involved in biosynthesis of sesame lignans, sesamin and sesamolin. In total, 9,347 putative functional genes from developing seeds were identified, which accounts for one third of total genes in the sesame genome. Further analysis of the uniESTs identified 1,949 non-redundant simple sequence repeats (SSRs).

**Conclusions:**

This study has provided an overview of genes expressed during sesame seed development. This collection of sesame full-length cDNAs covered a wide variety of genes in seeds, in particular, candidate genes involved in biosynthesis of sesame oils and lignans. These EST sequences enriched with full length will contribute to comparative genomic studies on sesame and other oilseed plants and serve as an abundant information platform for functional marker development and functional gene study.

## Background

Sesame (*Sesamum indicum *L.) belonging to the family pedaliaceae [1] is one of the most ancient self-pollinated oilseed crops. Sesame is one of oilseed crops with the highest oil content of up to 62.7% (average 52%) in seeds of sesame cultivars and accessions [2] when seed oil contents of other major oil crops peanut (*Arachis hypogaea*), oilseed rape (*Brassica napus*) and soybean (*Glycine max*) contain up to 54.0% (average 50%) [3], up to 46% (average 39%) [4] and up to 27.9% (average 20%) [5], respectively. Although recent intensive selection (imposed on natural variation or hybridization offspring) aided with high throughput Near-infrared spectroscopy for seed oil content determination with no seed damage can lead to more than 50% in oilseed rape, it is still lower than that having existed in sesame accessions for long time. Furthermore, sesame seed is highly nutritive in protein contents of the seed dry weight (25%) and has distinctive flavor. Sesame oil has excellent stability due to the existence of the natural antioxidants such as lignans sesamin, sesamol, sesamolin and sesaminol produced during seed development [6]. These natural antioxidants help to improve health qualities such as inhibiting *Δ*5-desaturase in mammals, enhancing vitamin E bioactivity, and inhibiting proliferation of human cancer cells [7-10]. Sesame lignans may also play a role in sesame resistance to insect pests and microbial pathogens [11].

The study on synthesis of high oil content and these antioxidants is poor, partly because of poor DNA and RNA sequence resources. At present, there are only about 3,000 sesame EST sequences available in GenBank [12]. Lack of sequence information and poor understanding of related synthetic biology have hindered genetic improvement on these economic characters as well as high unsaturated fatty acids contents in sesame. Thereby, our objective in this study was to build a large set of EST collection from developing seeds of sesame.

After construction of a normalized full-length cDNA library, we carried out large scale sequencing of the library and obtained more than 40,000 ESTs from developing seeds of high oil content Chinese sesame cultivar Zhongzhi 14, and further performed bioinformatics analysis on the ESTs with focus on those involved in oil and lignin biosynthesis. These sequence information will serve as the public-accessible fundamental resources for generation of molecular markers, genome annotation, gene discovery in sesame and comparative genomics study on oil contents of major oilseed crops.

## Results and discussion

### EST generation

**Figure 1:**
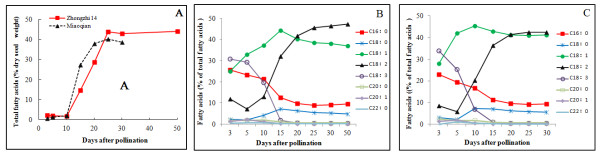
**Accumulation of oil and fatty acids in developing seeds of sesame**. The seeds of the sesame cultivars (*S. indicum*) Zhongzhi 14 and Miaoqian grew in two neighboring plots of a field in the institute farm. Fatty acid composition of oil was determined by the GC method. Oil contents (A) were calculated from the total fatty acids contents. B, C: Dynamic changes in fatty acid composition in developing seeds of sesame cultivars Zhongzhi 14 (B) and Miaoqian (C).

To understand accumulation of oil and lignan, and determine time of sampling developing seeds for EST sequencing, two sesame (*S. indicum*) cultivars Zhongzhi 14 with high oil content and its contrast Miaoqian with low oil content and early maturation were analyzed for accumulation of oil, fatty acids and lignans. The results indicated that the two cultivars have different oil contents but similar profiles of fatty acid compositions (Figure 1). Both cultivars started quick increase in oil contents from 10 days after pollination to maturation and oil accumulation reached their peak at about 25 days. Therefore sampling time was determined as 5, 20 and 30 days after pollination. Considering annual productivity (1321.5 kg per hectare of Zhongzhi 14, compared to 425 kg per hectare of Miaoqian) and oil content of seeds, Zhongzhi 14 was selected for construction of cDNA library enriched in full-length coding sequences [13] and EST sequencing.

The primary titer of the cDNA library was 1 × 10^6 ^cfu/mL with more than 90% recombinant clones revealed by X-Gal/IPTG screening and a small-scale quality assessment was performed prior to commencement of large-scale sequencing [13]. Plasmid DNAs were automatically prepared from the cDNA clones and sequenced from the 5' end by the Sanger method.

**Figure 2:**
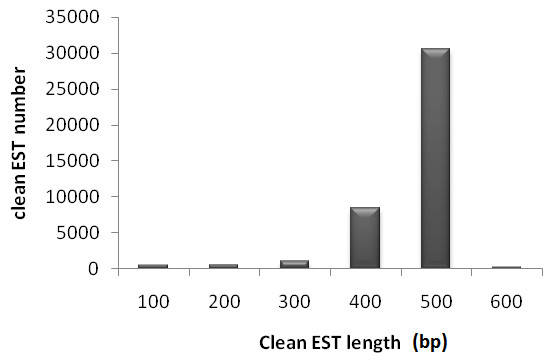
**Distribution of clean EST length of sesame**. The number of EST within different categories of trimmed sequence length is presented on the Y-axis. The number on the X-axis represents ranges of trimmed sequence lengths (101-200, 201-300, 301-400 bp, etc.). Clean EST length is cDNA length after removal of vector sequence and low quality sequences.

In total, 41,248 ESTs from single-pass 5' sequencing of 45,569 cDNA clones passed the quality control for high confidence base call with an average read length of approximately 570 bp (Figure 2). The overall sequencing success rate was 91%. The EST sequences generated in this study were deposited in GenBank with the accession numbers JK045130-JK086377. The GC content of the EST sequences was approximately 42.86%. Approximately 32.8% of the 41,248 sequences appeared twice or more times.

### Clustering of ESTs

After screening of low-quality DNA and trimming of vector sequences, Phrap program [14] was used to cluster the EST sequences and produce a uniESTs data set which comprised 4,713 tentative unique contigs (TUCs, see Additional file 1) and 27,708 tentative unique singletons sequences (TUSs, Table 1). The number of ESTs in the TUCs ranged from 2 to 92 and there are more than 65.5% contigs with 2 ESTs, 18.2% with 3 ESTs and 15.2% with 4-10 ESTs.

**Table 1:** Summary of ESTs from sesame

	Number	Percentage of uniESTs
Total ESTs sequenced	45569	-
Number of EST sequences with readable sequence	41248	-
Redundant sequences	13540	-
Number of uniESTs	32421	-
Number of singletons sequences	27708	85.5
Number of tentatively unique contigs	4713	14.5
uniESTs with significant matches against Nr	26131	80.6
uniESTs with non-significant matches against Nr	6289	19.4
uniESTs with significant matches to *A. thaliana*	12983	40

Among these uniESTs sequences, 80.6% had significant matches to sequences in the non-redundant protein database (Nr) with an E value cut-off equal or less than 10^-5^. Comparison of our uniESTs data set with the sesame EST sequence in GenBank using BLASTN demonstrated that only 349 uniESTs (1.1%) in our data set had significant matches to 903 sesame ESTs in GenBank (E-values ≤ 10^-5^) (Relevant information of these uniESTs was listed in Table 2).

**Table 2:** Frequency of sesame ESTs from the present study with significant similarities to sesame genes in the public database

Putative functions	EST number
11s globulin	32
Polyubiquitin	20
tubulin beta-1 chain	9
alpha tubulin 1	7
s-adenosylmethionine synthetase	7
alanine aminotransferase	6
rab11-family small gtpase	6
eukaryotic translation initiation factor 5a isoform iv	5
14-3-3 family protein	5
Actin	5
polyubiquitin containing 7 ubiquitin monomers	4
legumin-like protein	4
glyceraldehyde 3-phosphate dehydrogenase	4
aspartic proteinase	4
asparaginyl endopeptidase rep-2	4
nuclear antigen homolog	4
adenine nucleotide translocator	4
Calmodulin	4
2ss1_sesin2s seed storage protein 1 precursor (beta-globulin) (2s albumin storage protein)	4
glutathione s-transferase	4
kda oleosin	3
hydroxyproline-rich glycoprotein family protein	3
acr3 (act domain repeat 3)	3
heat shock protein 83	2
histone h3	2
high mobility group protein	2
20s proteasome beta subunit	2
26s proteasome non-atpase regulatory subunit 14	2
26s proteasome regulatory particle triple-a atpase subunit5a	2
3-hydroxy-3-methylglutaryl coenzyme a synthase	2
3-ketoacyl-thiolase acetyl-acyltransferase	2
40s ribosomal protein s8	2
60s ribosomal protein l11	2
adp-ribosylation factor	2
alcohol dehydrogenase	2
ap2 erebp transcription factor	2

**Figure 3:**
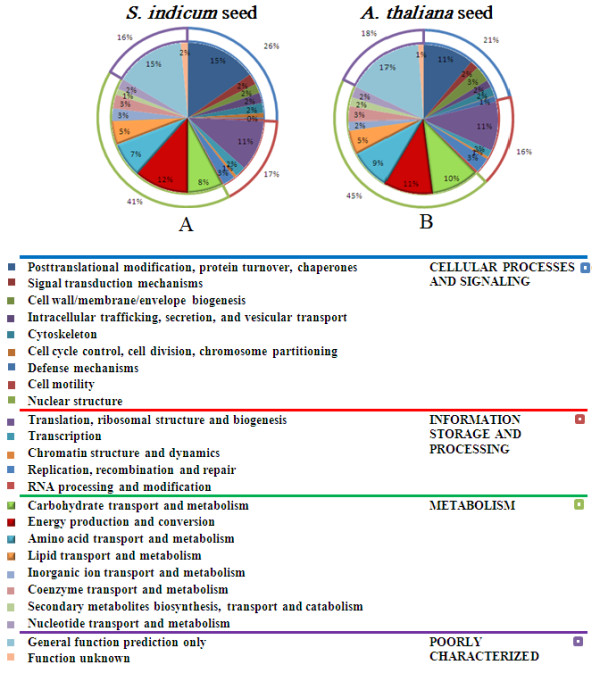
**Comparison of annotation of sesame ESTs (A) with those of *Arabidopsis *(B).** Two cDNA data sets were classified into functional groups by using the COG database; The colors of each functional group are indicated in the table. Graphs are outlined with multi-color frames which represent four subcategories: 'information storage and processing' (red), 'cellular processing and signaling' (blue), 'metabolism' (green) and 'poorly characterized' (purple). A: *S. indicum *seed COG annotaiton; B: *A. thaliana *seed COG annotation.

Comparison of the sesame uniESTs against the *A. thaliana *proteome database using BLASTX indicated 40% of these uniESTs with significant matches to those from *A. thaliana *(E-values ≤ 10^-5^). Based on identification of Clusters of Orthologous Groups of protein (COGs) [15] (Figure 3), 10,575 uniESTs (33.0%) of sesame seeds were assigned to COGs by BLASTX. The proportion pattern of each COG subcategory was similar between sesame and *A. thaliana *seeds [16] (Figure 3). In these two sets of cDNA sequences, only 1,360 sesame uniESTs are matched to *A. thaliana *seed genes (338 genes) [16] and 20% of these genes were involved in translation, ribosomal proteins synthesis, and 17% in posttranslational modification, protein turnover, chaperones. Only 1% of these genes were related to lipid transport and metabolism category.

### Protein coding regions

The full-length open reading frames (ORFs) in the uniESTs data set were identified. Homology search of the 32,421 uniESTs using BLASTX identified uniESTs with relatively high similarity (E-values ≤ 10^-5^) to known genes, where the sesame sequences, each had a start codon at a position similar to the protein sequence in GenBank, form a data set of putative full-length ORFs (44.9%). Furthermore, the codon usage table (Table 3) for the full-length sesame ORFs was generated using CODONW. The sesame (*S. indicum*) codon usage table containing 14,669 codons showed the GC content of the predicted coding region (46.7%) and the GC frequency at the third position (46.4%). The analysis was the first version of sesame codon usage table which was not presented in the Kazusa codon usage database http://www.kazusa.or.jp/codon/.

**Table 3:** Codon usage in *S. indicum*

		Second Letter																	
				
		U				C				A				G					
**First Letter**	**U**	**Phe**	**UUU**	**208**	**0.82**	**Ser**	**UCU**	**224**	**1.27**	**Tyr**	**UAU**	**192**	**0.67**	**Cys**	**UGU**	**113**	**0.9**	**U**	**Third Letter**
		**Phe**	UUC	301	1.18	Ser	UCC	259	1.47	Tyr	UAC	385	1.33	Cys	UGC	137	1.1	**C**	
		**Leu**	UUA	202	1.04	Ser	UCA	173	0.98	TER	UAA	49	1.20	TER	UGA	70	1.72	**A**	
		**Leu**	UUG	298	1.53	Ser	UCG	108	0.61	TER	UAG	3	0.07	Trp	UGG	95	1	**G**	
		
	**C**	**Leu**	CUU	192	0.99	Pro	CCU	151	0.87	His	CAU	151	1	Arg	CGU	231	1.63	**U**	
		**Leu**	CUC	216	1.11	Pro	CCC	165	0.96	His	CAC	150	1	Arg	CGC	180	1.27	**C**	
		**Leu**	CUA	70	0.36	Pro	CCA	275	1.59	Gln	CAA	280	1.05	Arg	CGA	48	0.34	**A**	
		**Leu**	CUG	190	0.98	Pro	CCG	100	0.58	Gln	CAG	254	0.95	Arg	CGG	72	0.51	**G**	
		
	**A**	**Ile**	AUU	399	1.27	Thr	ACU	357	1.55	Asn	AAU	261	0.97	Ser	AGU	121	0.68	**U**	
		**Ile**	AUC	420	1.33	Thr	ACC	275	1.19	Asn	AAC	275	1.03	Ser	AGC	175	0.99	**C**	
		**Ile**	AUA	126	0.4	Thr	ACA	212	0.92	Lys	AAA	520	0.99	Arg	AGA	222	1.57	**A**	
		**Met**	AUG	375	1	Thr	ACG	77	0.33	Lys	AAG	529	1.01	Arg	AGG	96	0.68	**G**	
		
	**G**	**Val**	GUU	331	1.39	Ala	GCU	396	1.51	Asp	GAU	412	1.13	Gly	GGU	402	1.54	**U**	
		**Val**	GUC	281	1.18	Ala	GCC	280	1.07	Asp	GAC	315	0.87	Gly	GGC	227	0.87	**C**	
		**Val**	GUA	161	0.68	Ala	GCA	247	0.94	Glu	GAA	532	1.16	Gly	GGA	333	1.28	**A**	
		**Val**	GUG	181	0.76	Ala	GCG	123	0.47	Glu	GAG	387	0.84	Gly	GGG	79	0.3	**G**	

Codon usage was calculated from 122 ORFs containing 14,669 codons.

### Gene ontology annotation

A total of 18,549 uniESTs were successfully annotated with Gene ontology (GO) terms using Blast2GO program [17]. An additional 3,079 sequences were then annotated using InterProScan program [18]. Overall, a total of 21,628 uniESTs were annotated with 111,600 GO terms distributing among the three main GO categories. 9,347 tentative unique genes (TUGs) representing 21,628 uniESTs across the various GO terms were examined with WEGO [19] (Table 4).

**Table 4:** Gene Ontology (GO) classifications of tentative unique genes from sesame according to their involvement in biological process, molecular function and cellular component

	uniESTs number	Percentage (%)	GO
**Cellular Component:**			
			
Extracellular region	**230**	2.5	GO:0005576
Cell	**5202**	55.7	GO:0005623
Virion	**20**	0.2	GO:0019012
Membrane-enclosed lumen	**273**	2.9	GO:0031974
Envelope	**191**	2.0	GO:0031975
Macromolecular complex	**999**	10.7	GO:0032991
Organelle	**2828**	30.3	GO:0043226
Extracellular matrix part	**5**	0.1	GO:0044420
Extracellular region part	**27**	0.3	GO:0044421
Organelle part	**931**	10.0	GO:0044422
Virion part	**19**	0.2	GO:0044423
Synapse part	**9**	0.1	GO:0044456
Cell part	**5056**	54.1	GO:0044464
Synapse	**19**	0.2	GO:0045202
Symplast	**2**	0.0	GO:0055044
**Biological Process:**			
			
Developmental process	**1475**	15.8	GO:0032502
Reproduction	**339**	3.6	GO:0000003
Obsolete biological process	**156**	1.7	GO:0008371
Reproductive process	**188**	2.0	GO:0022414
Immune system process	**42**	0.4	GO:0002376
Response to stimulus	**746**	8.0	GO:0050896
Multicellular organismal process	**870**	9.3	GO:0032501
Multi\-organism process	**151**	1.6	GO:0051704
Establishment of localization	**1278**	13.7	GO:0051234
Bological adhesion	**35**	0.4	GO:0022610
Metabolic process	**4319**	46.2	GO:0008152
Rhythmic process	**9**	0.1	GO:0048511
Viral reproduction	**17**	0.2	GO:0016032
Pigmentation	**1120**	12.0	GO:0043473
Locomotion	**29**	0.3	GO:0040011
Localization	**1299**	13.9	GO:0051179
Growth	**88**	0.9	GO:0040007
Cellular process	**4628**	49.5	GO:0009987
Cell killing	**1**	0.0	GO:0001906
Biological regulation	**1454**	15.6	GO:0065007
**Molecular Function:**			
			
Transcription regulator activity	**542**	5.8	GO:0030528
C atalytic activity	**3759**	40.2	GO:0003824
Binding	**3907**	41.8	GO:0005488
Antioxidant activity	**24**	0.3	GO:0016209
Chaperone regulator activity	**1**	0.0	GO:0030188
Translation regulator activity	**102**	1.1	GO:0045182
Structural molecule activity	**270**	2.9	GO:0005198
Transporter activity	**742**	7.9	GO:0005215

Under the category Biological Process, subcategories "cellular process" and "metabolic process" accounted for approximately 49.5% and 46.2% of the annotations for the TUGs, respectively, reflecting activeness of these processes. There are an overlap which is largely represented by the cellular metabolic process (37.1%) in the two subcategories and in the subcategory "metabolic process," primary metabolic process (36.4%), macromolecule metabolic process (27%) and biosynthesis (19.6%) account for large proportions, suggesting active metabolism of storage substances such as oil, lignan and proteins. In correspondence to these processes, in the main category Molecular Function, 41.8% of the TUGs annotations were grouped into the subcategory "binding" and 40.2% in the subcategory "catalytic activity" (Table 4).

### Analysis of ESTs involved in oil biosynthesis in developing sesame seeds

Comparative analysis indicated that the most redundant genes related to biosynthesis of fatty acid and oil in sesame included ketoacyl-CoA thiolase (KAT), pyruvate dehydrogenase complex (PDHC), plastidial long-chain acyl-CoA synthetase (LACS), stearoyl-ACP desaturase (SAD), acetyl-CoA carboxylase (ACC), ketoacyl-CoA reductase (KAR), oil-body oleosin (OBO), diacylglycerol acyltransferase (DGAT) etc. 496 uniESTs candidates were homologous to *Arabidopsis *oil-related genes (*Arabidopsis *Lipid Gene Database http://www.plantbiology.msu.edu) [20](Additional file 2). Of these, 71 uniESTs were mapped to the sesame genome sequence (Additional file 3 for DNA sequences of these genes) and just 12 genes like PDHC, OBO and LACS are homologous to those in the public sesame ESTs database (Additional file 2).

In the "fatty acid synthesis in the plastids pathway" [20], most of the key enzymes were found in our data set, such as the PDHC, ACC, plastidial acyl carrier protein (ACP) and KAR, except an important enzymes malonyl-CoA: ACP malonyltransferase (MCMT). After blasting against the whole sesame genome assembly, we found that most of these genes had lower copy numbers than those in *Arabidopsis*, except one gene encoding beta-ketoacyl-ACP synthase I (KAS I) which have 2 copies whereas the genomes of *Arabidopsis *has 1 copy (see Additional file 2 and Additional file 3). KAS I catalyzes the elongation of fatty acid synthesis for the carbon chain from C4 to C16, and played a crucial role in chloroplast division and embryo development [21]. The main components of sesame seed oil, oleic acid and linoleic acid, continuously increased up to about 80% of total fatty acids in the mature seeds (Figure 1). Some uniESTs related to these fatty acids elongation and desaturation in sesame were first reported in this study, such as putative full-length uniESTs encoding ketoacyl-ACP synthase II (KAS II) SAD and endoplasmic reticulum (ER) oleate desaturase (FAD2) which played important roles in the process of the conversion of 16:0-18:0 and desaturation.

Triacylglycerol (TAG) biosynthesis occurs at the ER and probably involves in reactions in the oil body as well [20]. Only four major genes, DGAT1, phosphatidylcholine: diacylglycerol acyltransferase (PDAT), OBO and caleosin, were detected, which involved in the last step of the TAG synthesis reaction and oil body formation in the pathway of synthesis and storage of oil. DGAT1 and PDAT have been known as the very important genes greatly affecting oil body formation and oil content [22]. OBO and caleosin were believed to facilitate mobilization of the TAG storage reserves [23,24].

Of 361 (3.9%) annotated uniESTs with putative transcription factor activity, 8 uniESTs were identified involved in oil accumulation, and they are homologous to *A. thaliana *transcription factor (TF) LEAFY COTYLEDON1 (AtLEC1), PICKLE (AtPKL) and WRINKLED1 (AtWRI1), respectively, suggesting their conservation and importance in transcriptional regulation of the fatty acid biosynthetic pathway. Of these TFs, putative sesame LEC1 (SiLEC1) and SiWRI1, like those in *Arabidopsis*, are single copies after blasting against whole sesame genome assembly (Additional file 3 for DNA sequences of these genes) and the sequence similarity of the two genes are 47% and 43% between sesame and Arabidopsis, respectively (Additional file 4). AtLEC1 positively regulates AtWRI1 and a large repertoire of fatty acid synthetic genes and several glycolytic genes [25]. Overexpression of AtWRI1 in Arabidopsis increased TAG content in transgenic plants [26] and overexpression of AtLEC1 and its orthologs in canola (*B. napus*) caused an increased fatty acid level in transgenic plants [27]. These homologs identified in sesame may have similar functions in oil biosynthesis.

### Analysis of ESTs involved in lignan biosynthesis in developing sesame seeds

**Figure 4:**
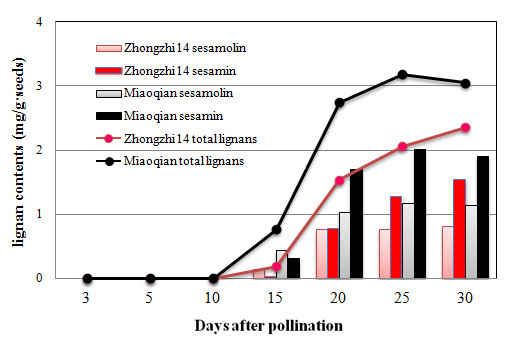
**The lignan contents of two sesame cultivars in different development stages**. The seeds of the sesame cultivars (*S. indicum*) Zhongzhi 14 and Miaoqian grew in two neighbouring plots of a field in the institute farm. Lignan content was determined by the HPLC method.

Two major oil-soluble lignans, sesamin and sesamolin [28] were quantitatively determined in the seeds of two cultivars (Zhongzhi 14 and Miaoqian) with contrast oil contents. Total lignan (sesamin and sesamolin) content was detectable 15 days after pollination and continuously accumulated until seed maturation (Figure 4). Interestingly sesamin content continuously increased from 15 days after pollination to seed maturation while sesamolin increased to its highest level at 20 days after pollination, and sesamin content was almost as twice as that of sesamolin at 30 days after pollination (Figure 4). In these two cultivars, it is clear that lignan formation was developmentally regulated [29,30]--lignan accumulation and seed development (maturity) keep the same pace. With seed development, the conversion ratio into sesamolin decreased whereas sesamin accumulation increased (Figure 4).

**Figure 5:**
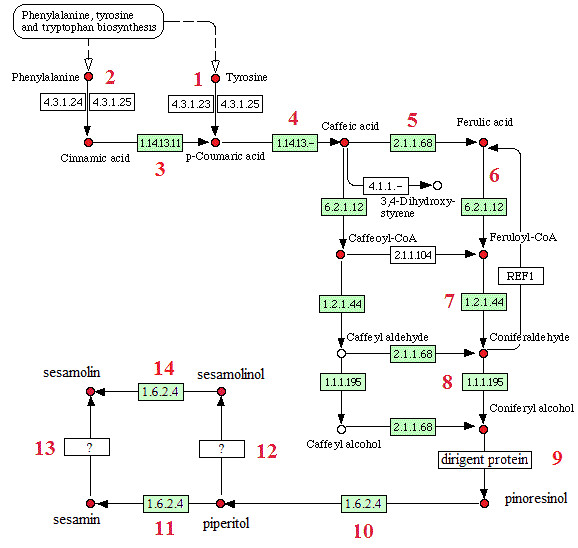
**Possible biosynthetic pathways for sesame lignans, sesamin and sesamolin**. The pathway map was constructed based on KEGG pathway map (see http://www.genome.jp/kegg-bin/show_pathway?map00940). Numbers correspond to individual reactions and serve to identify the respective enzyme in Additional file 5. Candidate gene found in the present sesame seed ESTs dataset was shown as green.

The antioxidant lignans, sesamin and sesamolin, are biosynthesized via the phenylpropanoid biosynthesis pathway [29,30]. In the pathway, tyrosine or phenylalanine is first converted into coniferyl alcohol which then undergoes stereoselective coupling to give pinoresinol, and further pinoresinol is metabolized in maturing seeds to piperitol, sesamin and sesamolin which are catalysed by O_2_/NADPH cytochrome P450s in the reactions 10, 11, and 14 where methylenedioxy bridges are formed (Figure 5) [29,30].

One hundred and 17 ESTs, corresponding to 7 EC numbers (EC1.1.1.195, EC 1.14.13.-, EC 1.14.13.11, EC 1.2.1.44, EC 1.6.2.4, EC 2.1.1.68, EC 6.2.1.12) (Figure 5), were identified possibly involved in the biosynthesis of sesame lignans during seed development according to KEGG blast result. Compared to the *Arabidopsis *database, 94 ESTs were homologous to 32 genes of *Arabidopsis*. In the GenBank database, there are just 12 sesame ESTs homologous to 4 uniESTs of our dataset encoding three enzyme genes (caffeate O-methyltransferase, COMT; cinnamyl-alcohol dehydrogenase, CAD; two P450 family members). Most of the EST candidates corresponding to enzymatic reactions in the biosynthetic pathway of sesame lignans, such as trans-cinnamate 4-monooxygenase (CA4H), oxidoreductases, COMT, 4-coumarate--CoA ligase (4CL), cinnamoyl-CoA reductase (CCR), CAD etc., were first identified from sesame (Additional file 5). The gene 4CL has the most abundant ESTs (45 putative uniESTs). There are 5 copies of the gene encoding COMT in the sesame genome (Additional file 6 for DNA sequences of these genes), but this gene is absent in the genomes of *Arabidopsis *and soybean. Surprisingly, neither phenylalanine ammonia-lyase nor tyrosine ammonia-lyase encoded ESTs were detected in the sesame seed ESTs. Thirty five ESTs of putative NADPH-cytochrome P450 oxidoreductase were identified, which involve in the important steps of sesame lignan production, including a gene encoding CYP81Q1 known for dual methylendioxy bridge formation on pinoresinol to produce sesamin *via *piperitol [31]. Because little information is available about enzymes for reactions from sesamin to sesamolin and from piperitol to sesamolinol [32], ESTs responsible for these enzymes was not able to be identified.

### EST-derived simple sequence repeat (SSR) markers

**Figure 6:**
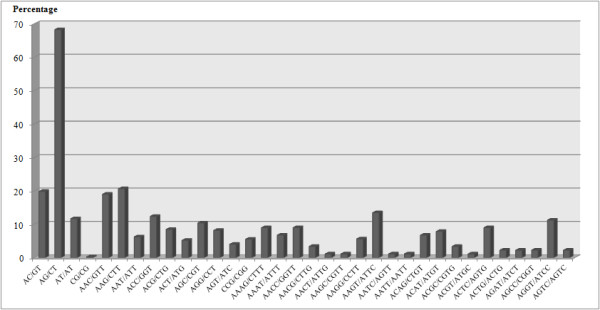
**The percentage distribution of the different SSR motifs (mono-, di-, tri-, tetra- and pentanucleotide)**.

Simple-sequence repeats (SSRs) have become one of major markers for population genetic analyses and marker-aided breeding [33,34]. A shortage of sesame molecular markers especially the EST-derived SSRs (EST-SSRs) have limited the efficiency of sesame molecular breeding [35]. Hence development of a large collection of EST-SSR will be very important source. In total, 1,688 uniESTs containing 1,949 non-redundant (NR) SSRs were identified from 32,421 uniESTs. The uniESTs represented about 17.428 Mb of genic sequences. A total of 226 sequences contain more than one SSR. The EST-derived NR SSRs were represented by mono-, di-, tri-, tetra- and pentanucleotide repeat motifs. This corresponds to an overall SSR density of approximately one SSR per 8.9 kb or one SSR-containing sequence in 5.2% of the NR EST sequences. About 8.3% of the SSRs identified were compound SSRs, which were defined as two neighbouring repeats located less than 10 nucleotides apart in a single sequence. The frequencies of the SSR motifs identified from 32,421 uniESTs were summarized in Table 5. Based on the distribution of SSR motifs, AG/CT motifs represented the most abundant dinucleotide repeat motifs (about 68%). The most common trinucleotide repeat motifs are, AAG/CTT (21%) and the most abundant tetranucleotide repeat motifs are the AAGT/ATTC (13%) (Figure 6).

**Table 5:** Frequency of non-redundant uniESTs-derived SSR motifs

Repeats	4	5	6	7	8	9	10	11	12	13	14	15	> 15	total
AC/GT	-	-	36	15	11	9	6	2	1					80
AG/CT	-	-	99	54	35	25	23	15	6	5	6	2	5	275
AT/AT	-	-	28	9	7	3								47
CG/CG	-	-	1											1
AAC/GTT	217	40	13	3					1					274
AAG/CTT	240	31	9	4	1	3		3	1	3	1		2	298
AAT/ATT	74	13	2	1										90
ACC/GGT	134	22	13	4	4	1								178
ACG/CTG	77	32	3	7	2		1							122
ACT/ATG	53	8	6	8										75
AGC/CGT	108	18	12	3	8	1								150
AGG/CCT	91	22	3		1	1								118
AGT/ATC	31	18	3			2	3						1	58
CCG/CGG	53	23	3		1									80
AAAG/CTTT	5	1	1	1										8
AAAT/ATTT	6													6
AACC/GGTT	8													8
AACG/CTTG	1	1		1										3
AACT/ATTG	1													1
AAGC/CGTT			1											1
AAGG/CCTT		5												5
AAGT/ATTC	12													12
AATC/AGTT	1													1
AATT/AATT	1													1
ACAG/CTGT	5		1											6
ACAT/ATGT	2		5											7
ACGC/CGTG	3													3
ACGT/ATGC	1													1
ACTC/AGTG	4	2	2											8
ACTG/ACTG		2												2
AGAT/ATCT	2													2
AGCC/CGGT			1	1										2
AGGT/ATCC	6	2	1	1										10
AGTC/AGTC	2													2
AGAGG/CCTCT	-		5											5
AGGGT/ATCCC	-	2												2
AACAAG/CTTGTT	-		1											1
AAGTAG/ATCTTC	-	1	1											2
ACCGAG/CTCTGG	-												1	1
AGCTCC/AGGTCG	-	1												1

Non-redundant SSR motifs were derived from 32,421 uniESTs.

## Conclusion

This study provided a set of ESTs enriched in full-length coding sequence generated from developing seeds. The number of these ESTs is more than 12.4 fold of the total number of entries for sesame in GenBank. From this set of ESTs, 9,347 putative functional genes from developing seeds were identified, accounting for one third of total genes in the genome. Most of the transcripts are the first representatives from sesame seeds. Most key enzymes and regulatory factors involved in fatty acid metabolism were found in our data, especially the enzymes responsible for the main unsaturated fatty acid synthesis, TAG synthesis reactions and oil body formation. Some conservative TFs significantly regulating oil synthesis were also identified. These provide a foundation for future comparative analysis of oil biosynthesis of different oilseeds. The uniESTs associated with biosynthetic enzymes of sesame lignans, sesamin and sesamolin, were identified and this information will be helpful for further studies on sesame lignan production. This large number of ESTs, half of them with full-length, have been used for sesame genome annotation in our sesame genome sequencing project and will be useful resource for the functional gene analysis and molecular marker development of traits such as contents of oil, fatty acids and lignan in sesame and for comparative genomics study of seed oil biosynthesis in different oilseed plants.

## Methods

### Plant materials

The seeds of the sesame cultivars (*S. indicum*) Zhongzhi 14 and Miaoqian from the Oil Crops Research Institute, Chinese Academy of Agricultural Sciences were sowed in two neighbouring plots of a field in the institute farm. Flowers were tagged in the early morning of the flower opening days. Developing capsules were harvested at 5-50 days or 30 days for early mature Miaoqian after pollination and seeds were immediately isolated and frozen in liquid nitrogen for RNA extraction or dried for seed chemical determination. Frozen seeds were stored at-70°C until use.

### Method for determination of sesame seed fatty acid

Fresh sesame seeds isolated from capsules were dried and ground for solvent extraction (soxlhet method). Fatty acid composition of oil was determined by the GC method according to the National Standards methods of China GB14489.3-1993-T, GBT 17376-2008, and GBT17377-2008 (the Official Methods and Recommended Practices of China). Heptadecanoic acid (17:0) was added to each sample as an internal standard. Oil contents were calculated from the total fatty acids contents.

### HPLC analysis of lignans

Lignans were determined by the HPLC method according to the National Standards methods of China NY/T 1595-2008. Sesamol was used as internal standards for calculating the percentage recovery of lignans.

### RNA extraction

Total RNA was isolated from developing seeds of sesame (cv. Zhongzhi 14) 5, 20, 30 days after pollination, respectively, and mixed in the same proportion, with TRIzol reagent (Gibco-BRL) according to the manufacturer's instruction. Poly A + RNA isolation was performed using PolyA Tract Isolation System (Promega, USA) according to the manufacturers' instructions.

### Generation of ESTs

Construction of a normalized cDNA library enriched in full-length sequences was reported [13]. The titer of unamplified cDNA library was about 1.0 × 10^6 ^cfu/mL. The percentage of recombinants was 100%. The results of gel electrophoresis showed fragments ranged from 700 bp to 2,000 bp, with an average length of 1,800 bp. The cDNA clones were cultured for plasmid DNA preparation manually. Automated cycle sequencing was performed by the Sanger method using T3 universal primer and BigDye Terminator (Applied Biosystems, USA) or ET Terminator (Amersham Pharmacia Bioscience, USA).

### Clustering analysis and annotation

Quality control of raw DNA sequences was performed by using Phred program [14]http://www.phrap.org/phredphrapconsed.html to remove sub-standard reads, the vector and adapter sequences, followed by EST-trimmer http://pgrc.ipk-gatersleben.De/misa/download/est_trimmer.pl to eliminate 3' polyA and 100 bp EST reads. Phrap program was used to cluster the overlapping ESTs into contigs. Groups that contained only one sequence were classified as singletons. The edited EST was translated into six reading frames and compared with the non-redundant protein database at the National Center for Biotechnology Information (NCBI) and the *Arabidopsis thaliana *Database at The *Arabidopsis *Information Resource (TAIR) http://www.arabidopsis.org/ using BLASTX program with the default settings (NCBI, ftp://ftp.ncbi.nlm.nih.gov/blast). BLASTN program was used to compare our nucleotide sequences with the sequences in the EST database in GenBank. BLASTX and BLASTN results with E-values cut-off 1e-10^-5 ^were treated as 'significant matches,' whereas ESTs with no hits or matches with E-values more than 10^-5 ^to proteins in GenBank were classified as 'no significant matches'.

### Assignment of GO terms

Gene Ontology (GO) terms were assigned to UniESTs by using Blast2GO [17] and summarized according to their molecular functions, biological processes and cellular components. The software performed BLASTX similarity search against the GenBank non-redundant protein (Nr) database, retrieved GO terms for the top 12 BLAST results and annotated the sequences based on defined criteria. A weightage based on the default Evidence Code Weights was also used to determine the GO terms annotated. In order to increase the number of sequences annotated by GO terms, additional information and GO terms were obtained by comparing the sequences to the InterPro database using the InterProScan tool [18]. More detailed functional annotations were performed by mapping tentative unique genes (TUGs) to the Gene Ontology Consortium structure which provided a structured and controlled vocabulary to describe gene products according to three ontologies: cellular components, biological processes and molecular functions. The distribution and percentage of TUGs in each of the GO terms were calculated. The GO terms were compared and visualized using WEGO http://wego.genomics.org.cn[19]. The percentage of 100 was defined as the total number of TUGs that were assigned GO terms. However, the percentages of the subcategories did not add up to 100% because many genes were involved in different classes of function and therefore annotated by multiple GO terms.

## Authors' contributions

SYL contributed to the conception, design and coordination of the study. HM and CHD were involved in the generation of sesame ESTs. TK analyzed the data and drafted the manuscript and SYL revised the manuscript. HC and XYD conducted determination of sesame seed fatty acids. YZZ and HYL prepared sesame cultivars materials. CBT assembled the sesame genome sequence and involved in the analysis of the sequence. All authors have read and approved the final manuscript.

## Supplementary Material

Additional file 1 **Tentative unique contigs of sesame**. Click here for file

Additional file 2 **A list of uniESTs involved in lipid metabolism and relevant homologies of Arabidopsis and sesame genes**. Click here for file

Additional file 3 **The blat and blast result of the uniESTs involved in lipid metabolism to S. indicum genome sequence and putative genes**. Click here for file

Additional file 4 **Phylogenetic analysis of the two transcriptional factor WRI1 and LEC1 Proteins**. The phylogenetic tree was built by MEGA5 [36]. Click here for file

Additional file 5 The blat [37] and blast result of the uniESTs involved in lignans metabolism to S. indicum genome sequence and putative genes. Click here for file

Additional file 6 Candidate ESTs involved in biosynthetic pathways for sesame lignans, sesamin and sesamolin, and relevant homologies of Arabidopsis and soybean genes. Click here for file
