# Supplementary figures and images for: Analysis of expression sequence tags from a full-length-enriched cDNA library of developing sesame seeds (Sesamum indicum)
Source: BMC Plant Biol. 2011 Dec 24;11:180. doi: 10.1186/1471-2229-11-180 (PMC3311628; doi:10.1186/1471-2229-11-180)

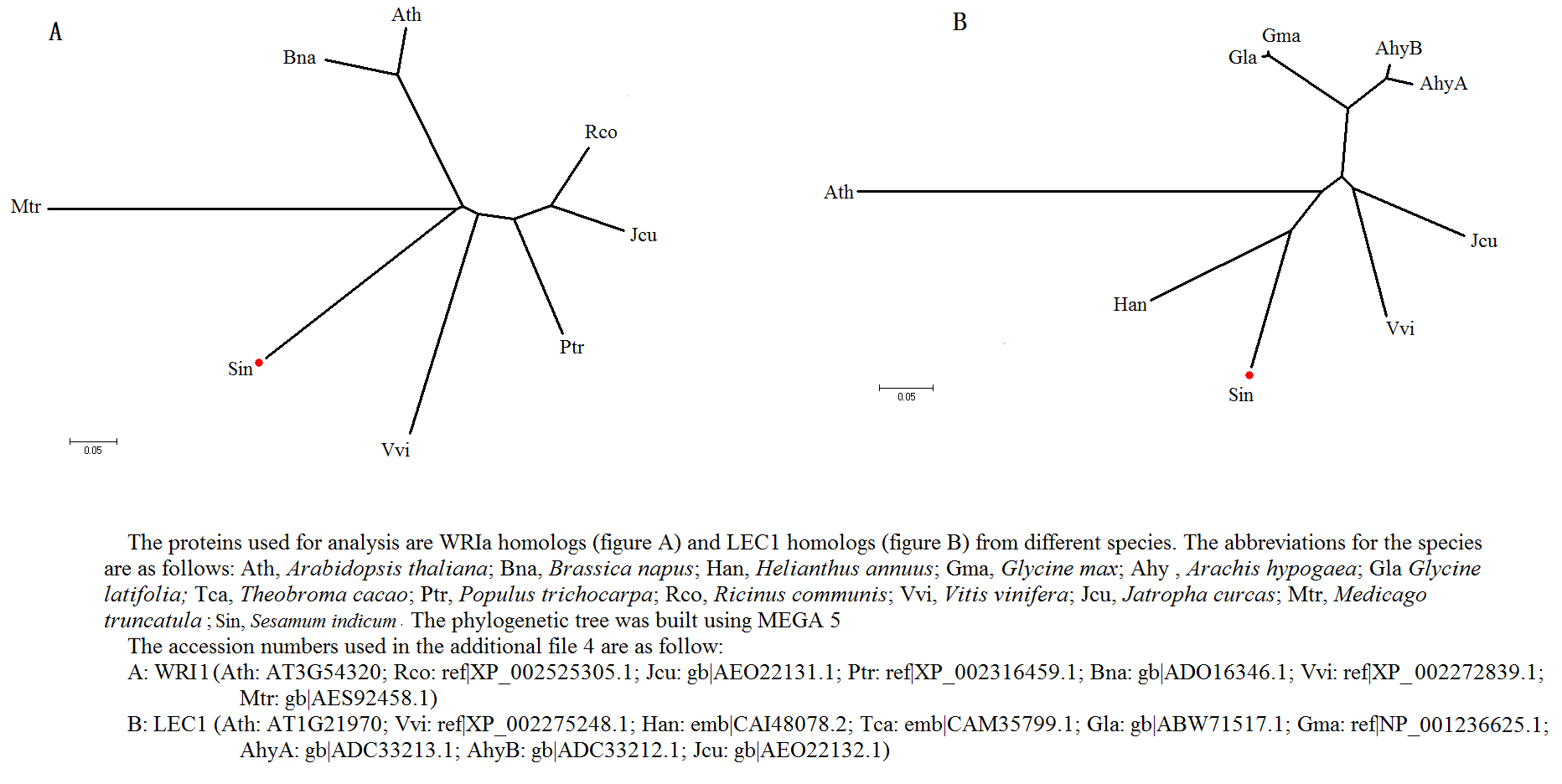

Supplement: Additional file 4 — Phylogenetic analysis of the two transcriptional factor WRI1 and LEC1 Proteins. The phylogenetic tree was built by MEGA5 [36]. [file 1471-2229-11-180-S4.TIFF]
